# High levels of unreported intraspecific diversity among RNA viruses in faeces of neonatal piglets with diarrhoea

**DOI:** 10.1186/s12917-019-2204-2

**Published:** 2019-12-05

**Authors:** Martí Cortey, Ivan Díaz, Anna Vidal, Gerard Martín-Valls, Giovanni Franzo, Pedro José Gómez de Nova, Laila Darwich, Héctor Puente, Ana Carvajal, Marga Martín, Enric Mateu

**Affiliations:** 1 Departament de Sanitat i Anatomia Animals, Facultat de Veterinària, Universitat Autònoma de Barcelona, 08193 Cerdanyola del Vallès, Spain; 2 IRTA, Centre de Recerca en Sanitat Animal (CReSA, IRTA-UAB), Campus de la Universitat Autònoma de Barcelona, 08193 Cerdanyola del Vallès, Spain; 3 Department of Animal Medicine Production and Health (MAPS), University of Padova, Viale dell’Università 16, 35020 Legnaro, PD Italy; 4 Departamento de Sanidad Animal, Facultad de Veterinaria, Universidad de León, Campus de Vegazana s/n, 24071 León, Spain

**Keywords:** RNA virus, Piglets, Diarrhoea, Next-generation sequencing

## Abstract

**Background:**

Diarrhoea is a major cause of death in neonate pigs and most of the viruses that cause it are RNA viruses. Next Generation Sequencing (NGS) deeply characterize the genetic diversity among rapidly mutating virus populations at the interspecific as well as the intraspecific level. The diversity of RNA viruses present in faeces of neonatal piglets suffering from diarrhoea in 47 farms, plus 4 samples from non-diarrhoeic piglets has been evaluated by NGS. Samples were selected among the cases submitted to the Veterinary Diagnostic Laboratories of Infectious Diseases of the *Universitat Autònoma de Barcelona* (Barcelona, Spain) and *Universidad de León* (León, Spain).

**Results:**

The analyses identified the presence of 12 virus species corresponding to 8 genera of RNA viruses. Most samples were co-infected by several viruses. *Kobuvirus* and *Rotavirus* were more commonly reported, with *Sapovirus*, *Astrovirus* 3, 4 and 5, *Enterovirus G*, *Porcine epidemic diarrhoea virus, Pasivirus* and *Posavirus* being less frequently detected. Most sequences showed a low identity with the sequences deposited in GenBank, allowing us to propose several new VP4 and VP7 genotypes for *Rotavirus B* and *Rotavirus C*.

**Conclusions:**

Among the cases analysed, Rotaviruses were the main aetiological agents of diarrhoea in neonate pigs. Besides, in a small number of cases *Kobuvirus* and *Sapovirus* may also have an aetiological role. Even most animals were co-infected in early life, the association with enteric disease among the other examined viruses was unclear. The NGS method applied successfully characterized the RNA virome present in faeces and detected a high level of unreported intraspecific diversity.

## Background

Diarrhoea in neonatal piglets is a common problem in many pig herds. Usually, this condition is seen either in the offspring of gilts or as an epidemic problem affecting litters from sows of any parity. In the first case, the problem is usually attributed to an inadequate acclimation protocol for gilts while in the latter, the most often cause is the introduction of a new enteric pathogen. These patterns reflect the immune status of sows against the causing agents and the transfer of colostral and lactogenic immunity to piglets. The agents involved in neonatal diarrhoea of piglets are diverse and include bacterial (*Escherichia coli, Clostridium perfringens*, etc.) and viral pathogens (coronaviruses, rotaviruses, etc.). Less commonly neonates can suffer from diarrhoea caused by parasites [1]. Most of the viruses causing outbreaks of diarrhoea in neonate piglets are RNA viruses such as *Porcine epidemic diarrhoea virus* (*PEDV*), *Transmissible gastroenteritis virus* (*TGEV*), *Porcine deltacoronavirus* (*PDCoV*) or *Rotavirus A, B, C* or *H* (*RVA*, *RVB*, *RVC*, *RVH*) [2–9]. Among them, *Rotavirus A*, *B* and *C* are a major cause of diarrhoea in pigs worldwide, having a significant impact in health and productivity, plus the potential of zoonotic transmission to humans [7]. The virus is transmitted by the faecal-oral route and large number of viral particles is excreted during *Rotavirus* infection. In contrast, other RNA viruses including *Kobuvirus, Astrovirus, Sapovirus*, *Sapelovirus*, *Teschovirus,* and *Torovirus,* have been detected in pig faeces but its role as causative agents of neonatal diarrhoea has not so far been fully elucidated [10–14]. For instance, porcine *Kobuvirus* (*KobuV*) has been found frequently (16–99%) in faecal samples of pigs worldwide [15]. In some cases, the presence of the virus was associated with diarrhoea, but in others no significant differences between infection rates in healthy or diarrhoeic animals was found. Frequently *KobuV* were present concomitantly with other viruses making difficult a precise assessment of its role as agent of disease [12, 16].

Next Generation Sequencing (NGS) allows for an in-depth characterization of the genetic diversity among rapidly mutating virus populations by directly sequencing viral strains at the interspecific as well as the intraspecific level [17, 18]. The technology has been successfully applied to identify the diversity in the viral genome of pigs’ faeces [15, 19–21]. The aim of the present study is to explore the diversity of RNA viruses present in faeces of neonatal piglets suffering from diarrhoea in Spain, applying a tailor-made NGS protocol using the total RNA extracted, without any amplification step, where the results obtained represent not only the RNA viruses present in a sample, but also their relative abundances.

## Results

**Fig. 1:**
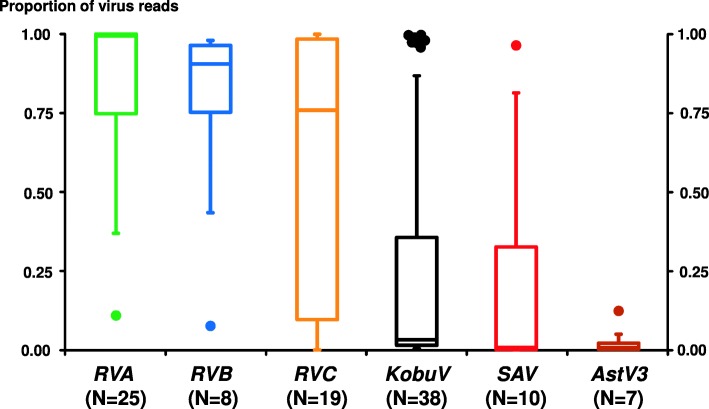
The box-and-whisker plots show, for the 47 samples from diarrhoeic animals, the distribution of the proportion of reads for a given mammalian viral species over the total number of mammalian viral reads (viral reads in a sample/total viral reads). Minimum, maximum, median, 25 and 75 quartiles are shown. Only virus showing read proportions larger than 0.001 are represented. Virus abbreviations: *Rotavirus A, RVA; Rotavirus B, RVB; Rotavirus C, RVC; Kobuvirus, KobuV; Sapovirus, SAV; Astrovirus 3, AstV3*

The results reported among the 47 diarrhoeic samples analysed include representatives of 12 virus species corresponding to 8 genera of RNA viruses (Additional file 1): *Kobuvirus*, *Rotavirus* (*RVA*, *RVB* and *RVC*), *Sapovirus (SAV)*, *Mamastrovirus* (*Porcine Astrovirus* types 3 - *AstV3* -, 4 - *AstV4* - and 5 – *AstV5* -), *Alphacoronavirus* (*PEDV*), *Enterovirus* (Enterovirus G, *EntVG*), *Pasivirus* (*PasiV*) and *Posavirus (PosaV)*. The most commonly reported RNA virus was *KobuV*, followed by *RVA*, *RVC*, *SAV, RVB* and *AstV3* (N values in Fig. 1). Among the 9 diarrhoeic samples where a single virus was detected, *RVA* was present in 5 samples and *KobuV* was present in 4. Thirty-eight samples (81%) presented more than one virus and in one sample seven RNA viruses were identified: *KobuV*, *RVC*, *AstV* 3, 4 and 5, *SAV* and *PEDV*. *Rotavirus* (either *RVA*, *RVB* or *RVC*) plus *KobuV* was the combination present in 12 out of the 14 (86%) samples infected with 2 viruses. *RVA*, *RVB*, *RVC* or a combination of two or three *Rotavirus* were also present in 41 out of the 47 (87%) diarrhoeic samples analysed. For the 6 *Rotavirus*-negative samples *KobuV* was present in all 6; being the single virus present in 4 of them. In the other two samples *Astrovirus* 3, 4, and 5, *SAV*, *PasiV*, *PosaV* and *EntVG* were detected (Additional file 1).

**Fig. 2:**
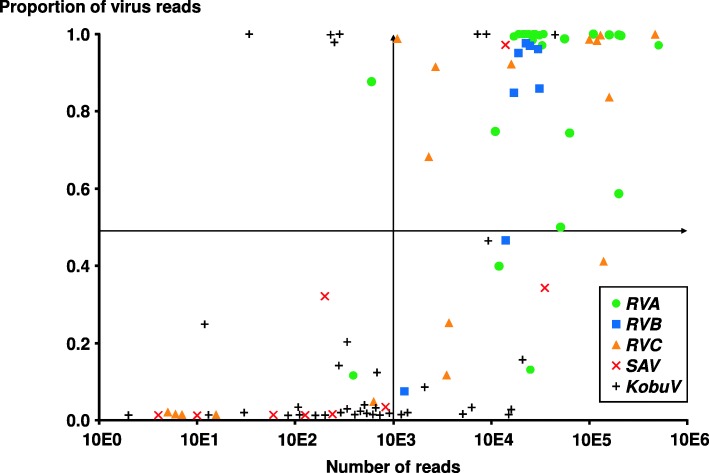
Scatter plot graph representing the number of viral reads indexed per virus and per sample (X-axis), versus the proportion that those viral reads represents over the total number of mammalian virus reads indexed (Y-axis) in each one of the 47 diarrhoeic animals analysed. Only *Rotavirus A*, *B* and *C*, *KobuV* and *SAV* were considered

The NGS approach used allowed the comparison of the relative frequencies of each viral species in the examined samples. Thus, when the number of reads for a given virus was compared to the total number of reads attributable to a mammalian RNA virus in the sample, it was evident that Rotaviruses, particularly *RVA* and *RVB*, clearly outnumbered any other virus that could be present (Fig. 1). In only nine cases, *KobuV* or *SAV* reads were predominant among viral reads (8 and 1 cases, respectively). In all 47 cases, one virus represented at least 45% of the total viral reads. Interestingly, when mixed rotavirus infections were detected (*RVA*-*RVC* or *RVB-RVC*), the proportion of one virus clearly predominated against the other. When coupled with the viral load, the relative frequencies per virus (Fig. 2) showed that for a majority of cases (40/47, 85.1%), the predominant virus in the sample represented read proportions higher than 0.5 and viral reads figures larger than 10E3. Nearly all of them (36/40, 90.0%) corresponded to *Rotavirus* – either *RVA* (21), *RVB* (6) or *RVC* (9) –, with *KobuV* and *SAV* being much less frequent (3 and 1 cases, respectively). Besides, few cases (4 *KobuV* and 1 *RVA*) represented simultaneously high proportions with viral reads below 10E3.

In the four non-diarrhoeic samples, the number of viral reads obtained and indexed was lower compared to those of the diarrhoeic samples, none of the mammalian virus detected surpasses 10E0 to 10E1 reads (Additional file 1). In contrast, a large number of reads were indexed against RNA viruses of plants (i.e. *Fabavirus*, *Bromovirus*, *Fijivirus*, *Luteovirus*) and fungi (i.e. *Hypovirus*, *Alphapartivirus*).

The filtering results included both whole genome (complete) and partial (less than 90% of the genome covered) sequences of the virus listed above (Additional file 1). Despite *KobuV* was more commonly reported than *RVA*, the number of full-length genome sequences obtained was higher in *RVA* (*n* = 23) than in *KobuV* (*n* = 17). The full-length sequences obtained – 17 *KobuV*, 23 *RVA*, 11 *RVC*, 8 *RVB*, 4 *SAV* and 2 *AstV*3 – were deposited in GenBank with the Accession Numbers MH238075 to MH238338, MK936372 to MK936426, MK953017 to MK953236, and MK962320 to MK962342. Considering the segmented nature of the *Rotavirus* genome, every segment for every genome was uploaded separately. For the remaining RNA viruses, every complete genome was uploaded in a single file.

**Fig. 3:**
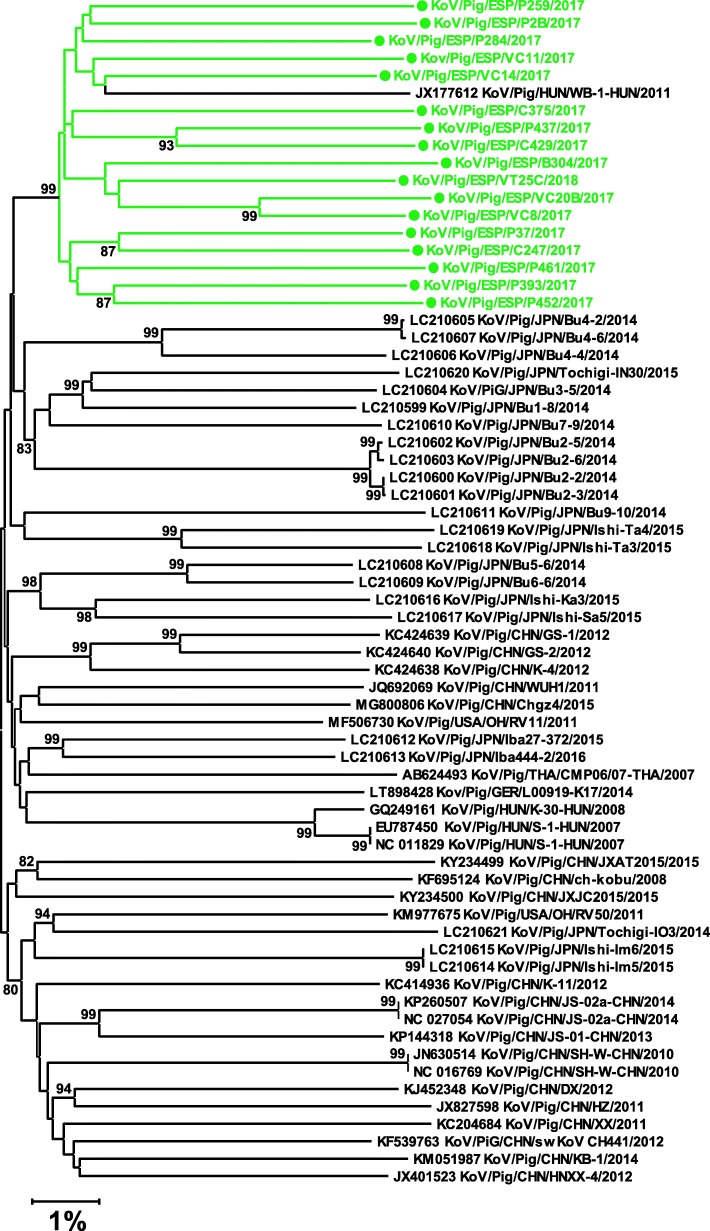
Neighbor-joining phylogenetic tree based on the p-distance among the nucleotide sequences of the complete genomes for *Kobuvirus*. Along the branches, percentage of bootstrap values based on 10,000 replicates. Only values equal or larger than 80% are shown. The dataset contains the 51 complete *Kobuvirus* genomes available in GenBank plus the 17 genomes – marked in green and with a circle – reported in this work

Regarding the phylogenetic analysis of *KobuV* (Fig. 3), all Spanish sequences formed a monophyletic cluster where a Hungarian sequence was also included. The average nucleotide identity among Spanish isolates was 90.0%. It is worth noting that the depth of the terminal branches in the phylogenetic tree was very high compared to the inner branches, indicating a low number of shared mutations.

**Fig. 4:**
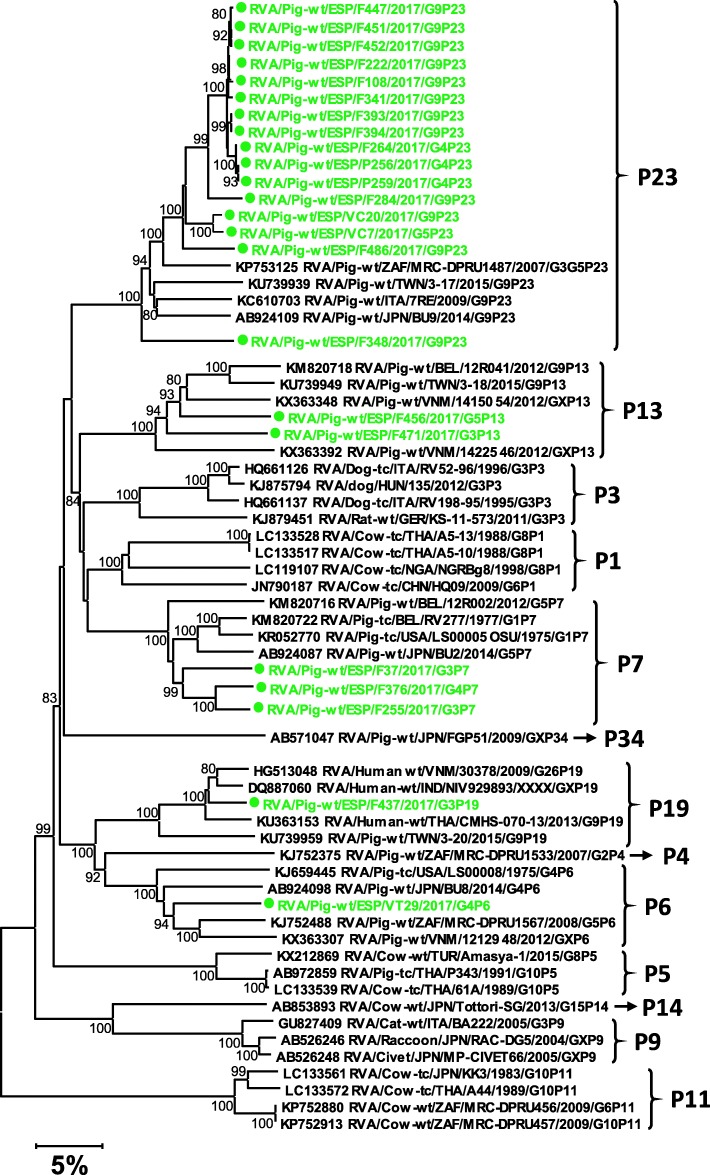
Neighbor-joining phylogenetic tree based on the p-distance among the nucleotide sequences of the VP7 segment for *Rotavirus A*. Along the branches, percentage of bootstrap values based on 10,000 replicates. Only values equal or larger than 80% are shown. The dataset contains 41 reference sequences representing RVA strains isolated in different species, included for comparison purposes, plus the 23 sequences – marked in green and with a circle – reported in this work. The division of genotypes is based on the 80% nucleotide cut-off values proposed by the RotaC 2.0 genotyping tool [23], and the RVA genotype determination tool of Virus Pathogen Resource [24]

**Fig. 5:**
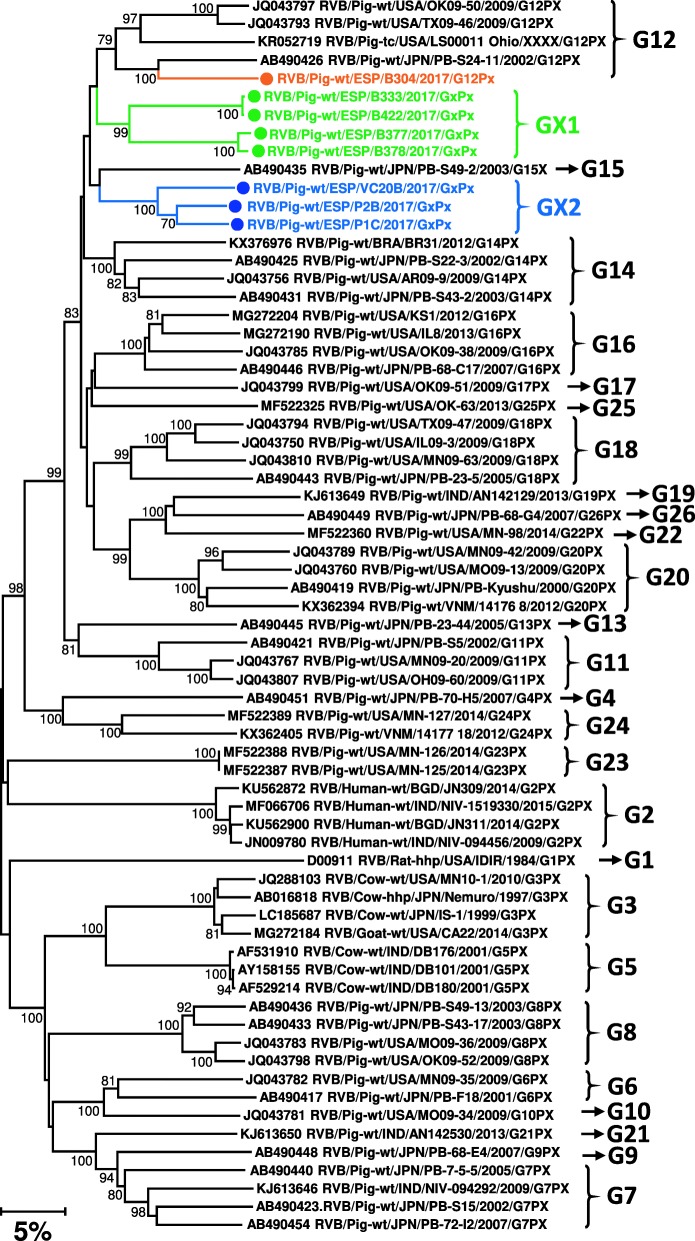
Neighbor-joining phylogenetic tree based on the p-distance among the nucleotide sequences of the VP7 segment for *Rotavirus B*. Along the branches, percentage of bootstrap values based on 10,000 replicates. Only values equal or larger than 80% are shown. The dataset contains 58 reference sequences representing the 26 *RVB* VP7 genotypes described plus the 8 sequences – marked with a circle – reported in this work. The division of genotypes is based on the 80% nucleotide cut-off value proposed by [25]; accordingly, two new genotypes (labelled as GX1 and GX2 in the Figure) are tentatively proposed

The genotyping of the 23 *RVA* genomes obtained indicated that the most common combination was G9P [22] (12 cases; 52%), with G4P [22] (3 cases; 13%) and G3P [7] (2 cases; 9%) being much less frequent. The genotypes G3P [13], G3P [19], G4P [7], G5P [13], G5P [22] and G4P [6] were reported only once (Fig. 4). The nucleotide identities reported for the *RVA* segments compared to the existing GenBank sequences ranged between 89 and 98%. In contrast, for the 8 *RVB* complete genomes characterized in this work (Fig. 5), the overall nucleotide identities (among the 11 segments of the *RVB* genome) with the closest GenBank sequences ranged from between 74 and 90%. In fact, according to the proposed nucleotide cut-off values for VP7 (80%), two undescribed VP7 genotypes were reported in nearly all (7 out of 8) the *RVB* positive samples. Similarly, for VP4, 3 samples could harbour a new *RVB* genotype (tree not shown). The *RVB* genotypes reported and its frequency would be: GX2P [4] (3, 37.5%), GX1P[X] (2, 25%), GX1P [4] (2, 25%), and G12P[X] (1, 12.5%).

**Fig. 6:**
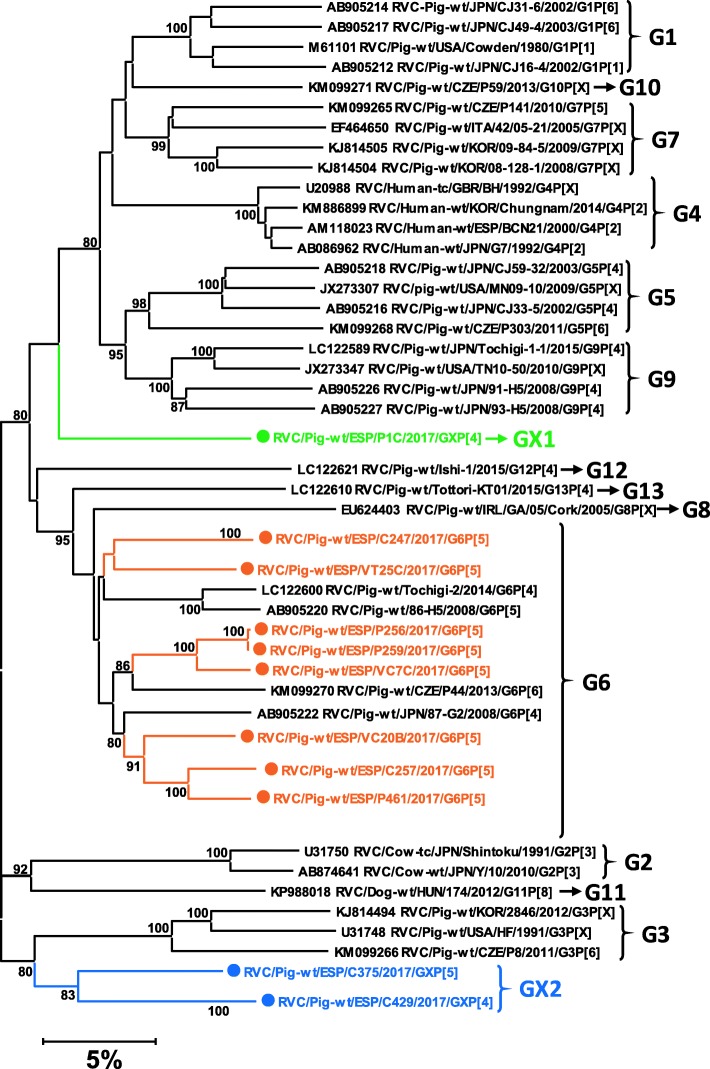
Neighbor-joining phylogenetic tree based on the p-distance among the nucleotide sequences of the VP7 segment for *Rotavirus C*. Along the branches, percentage of bootstrap values based on 10,000 replicates. Only values equal or larger than 80% are shown. The dataset contains 34 reference sequences representing the 11 *RVC* VP7 genotypes described plus the 17 sequences – marked with a circle – reported in this work. The division of genotypes is based on the 85–86% nucleotide cut-off value proposed by [26]; accordingly, two new genotypes (labelled as GX1 and GX2 in the Figure) are tentatively proposed

For *RVC* (Fig. 6), the nucleotide identities with the sequences available in GenBank ranged between 83 and 91%. The most common genotype was G6P [5] (8 samples), while the remaining 3 genomes would present two new *RVC* VP7 genotypes in combination with P [4] – one with GX1P [4] and one with GX2P [4] – and one GX2P [5].

**Fig. 7:**
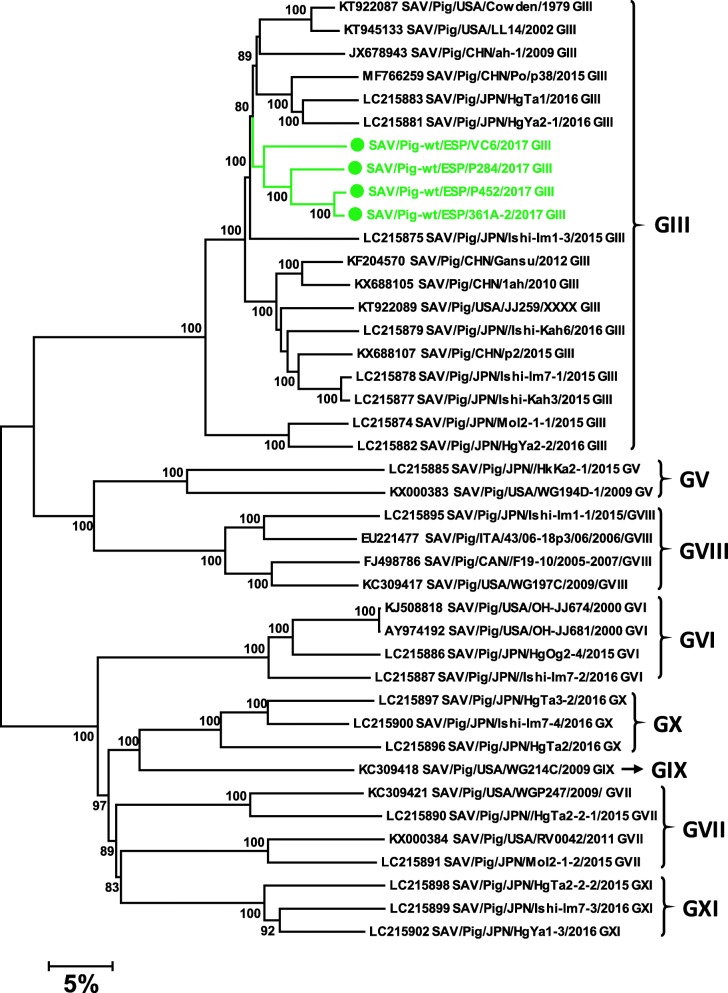
Neighbor-joining phylogenetic tree based on the p-distance among the nucleotide sequences of the complete genomes of *Porcine Sapovirus*. Along the branches, percentage of bootstrap values based on 10,000 replicates. Only values equal or larger than 80% are shown. The dataset contains 37 reference genomes representing the 8 *Sapovirus* genogroups described so far in swine plus the 4 complete sequences – marked with a circle – reported in this work. The division of genotypes is based on the classification proposed by [27]

**Fig. 8:**
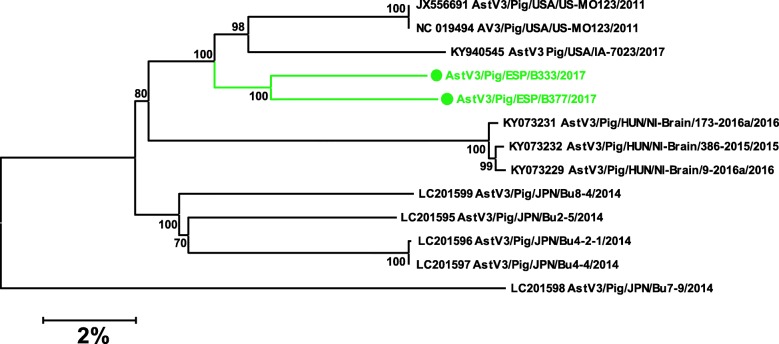
Neighbor-joining phylogenetic tree based on the p-distance among the nucleotide sequences of the complete genomes of *Porcine Astrovirus 3*. Along the branches, percentage of bootstrap values based on 10,000 replicates. Only values equal or larger than 80% are shown. The dataset contains the 11 complete genomes available so far in swine plus the 2 complete sequences – marked with a circle – reported in this work

For other viruses, nucleotide identities with the closest sequences deposited were low: *AstV3* (91%), *AstV4* (89%), *SAV* (87%), *EntVG* (82%) and *PasiV* (86%) except for *PosaV* (96%), *AstV5* (97%) and *PEDV* (99%) which were very similar to other European sequences reported. Figures 7 and 8 show the phylogenetic trees for *SAV* and *AstV3*, respectively.

The de novo assembler and virus detection analyses performed did not detect contigs that could be related to the presence of an unknown RNA virus, as all of them where positively indexed against the Reference RNA sequences dataset and/or the RefSeq Genome Database available at the BLAST resource of the NCBI website.

## Discussion

Diarrhoea is the most common infectious cause of neonatal death in pigs [28]. Prevention of this condition can be achieved by immunization of the sow with the aim of ensuring transfer of immunoglobulin A in colostrum and milk. Indeed, outbreaks of infectious diarrhoea in neonates are associated to the lack of specific immunity in the sow.

Classically, infectious neonatal diarrhoea has been seen as a problem caused by single aetiological agents. However, with the development of molecular techniques, a growing number of evidences indicated that many agents can be found in the faeces of affected animals [19, 21]. The problem now is to distinguish which of those agents primary cause diarrhoea, which others act as secondary or associated agents and which of them are part of the enteric microbiome without involvement in the disease.

In this work, we examined the RNA virome present in faecal samples from 47 cases of diarrhoea plus four non-diarrhoeic negative controls in which specific bacteriological agents were excluded. The approach taken using NGS did not include any previous enrichment or PCR step; hence, the results obtained represented not only the RNA viruses present in a sample analysed but also their relative abundances. This may contribute to our understanding of the role of each virus in a given case.

In the samples examined, the predominance of Rotaviruses reads reinforce the notion of these viruses as primary agents of neonatal diarrhoea; although, occasionally, *KobuV* and *SAV* may have a role in this process. The examination of the relative proportions of viral reads versus the number of reads showed that for any given case always one virus predominated, representing more than 45% of the mammalian viral reads obtained. This value can be tentatively proposed as a cut-off for the assignment of an etiological agent. In contrast, for all the other viruses examined, the relative abundance of their genomes would be more consistent with a subclinical infection. Whether this could be the consequence of a limited virulence, because of some level of passive immunity, or other causes, cannot be addressed in the present study.

Since the NGS filtering method used in the present study could only detect known agents, additional analyses to screen for potential viral motifs were performed. The de novo assembly and virus detection analyses were not able to identify any contig that could be related to the presence of an unknown RNA virus.

Interestingly, in the non-diarrhoeic samples analysed the number of mammalian reads was very scarce, but a higher number of plant and fungi viruses was reported. This is somewhat surprising, since the examined animal were suckling piglets (less than 1 week of age) that do not eat feedstuff. It is difficult to explain the origin of those viruses, since samples were taken from the rectum and thus, environmental contamination is little likely.

Our results agree with a number of studies that reported different Rotavirus species as agents of neonatal diarrhoea (reviewed in [7]). One interesting observation from the present study is that coinfections or *RVA-RVC* and *RVB-RVC* was very common, suggesting the need for simultaneous testing all three agents in cases of neonatal diarrhoea. Regarding *KobuV*, our results also agree with an increased prevalence of this agent observed in cases of diarrhoea in suckling piglets worldwide: Brazil [22], Korea [29] and Vietnam [30]; despite several studies have observed non-significant differences in *KobuV* infection between diarrheic and healthy piglets in several European countries [12, 16, 31]. As mentioned above, two different patterns were observed in cases where *KobuV* was present: one with high proportion of *KobuV* reads and high number of viral reads, and others where these two circumstances were not fulfilled. The first pattern could correspond to clinical cases caused by *KobuV*, while the second could correspond to secondary or concomitant infections by this agent. A similar pattern would apply to *SAV*. Regarding the other agents, since they were always in combination and their reads were not preeminent, its role must be seen as secondary at best. For instance, no differences in *Astrovirus* prevalence between diarrheic and non-diarrheic piglets were reported [32, 33], nor for *Enterovirus* prevalence between healthy and diarrheic pigs [30]. Interestingly, *PEDV* was seldom found in our samples and when found only traces could be detected. Prior to this, a higher frequency for this pathogen could be expected. However, while PEDV has caused recently severe epidemics in America [18], in Europe the incidence seems to be much lower [34]. Of note is the total absence of TGEV, a virus which was widespread in Europe, but has consistently declined, or even disappeared, after the apparition and fast spread worldwide in the nineties of *Porcine Respiratory Coronavirus* [35].

In any case, it is somewhat surprising to detect that many different viruses (up to seven in a single sample) in so young animals, even though the pattern was already observed in diarrhoeic piglets [21]. While it is easy to understand that the introduction of a new enteric virus in the farm will result in its rapid spread and the eventual development of an epidemic, it is more difficult to understand how so many viruses can be present in the maternities without generating a herd immunity resulting in colostral and lactogenic protection of suckling piglets. One possibility is that, most often, maternal immunity could be enough to limit the replication of those agents but not to completely prevent the infection; namely only provided partial immunity. In other cases, on farms reporting disease even in sows, *RVA* was present. This is compatible with its assumed role as primary agent. We have recently described the introduction of a new *RVA* strain that rapidly spread across pig farms in Spain and that was also present in the samples of this study as well [36].

It is worth noting that the phylogenetic analyses for all the examined viruses indicated the existence of local clusters except for *RVA* (discussed above). This, together with the depth of the corresponding branches in the trees, suggested a long evolutionary history on a local basis. Certainly, we cannot discard the existence of other clusters for any of the reported viruses. However, the detection of several new genotypes for *RVB* and *RVC* plus the relative low nucleotide identity with other available sequences in GenBank reinforces the idea of local evolution at least for these two viruses. Moreover, with the results obtained we propose to define for *RVB* one new VP4 (P [6]) and two new VP7 (G27 and G28) genotypes; as well as genotypes G14 and G15 for *RVC*. The geographical pattern observed in the *RVB* phylogenetic trees agrees with the notion that *RVB* genotypes may be specific for host species and region [25]. Regarding *KobuV*, no differences in the clustering pattern were observed between those strains coming from cases in which *KobuV* was predominant or not. This suggests that phylogenetic clustering probably is not predictive of virulence for this virus and most probably, other causes (i.e. immunity) are more relevant with regards to the clinical expression of the disease. Similarly, for *SAV* no differences were seen, and all isolates clustered together within genogroup III, the most commonly reported porcine *SAV* [37].

## Conclusions

The results of this study suggested that based on the abundance among the cases analysed, Rotaviruses were the main RNA viruses involved in neonatal diarrhoea in pigs. Although *Kobuvirus* was the most common virus detected, probably it was the primary agent of diarrhoea only in a small number of cases. Similarly, *Sapovirus* would be responsible of a single diarrhoea case. For the other examined viruses, the results indicated that many animals were infected in early life but the association with enteric disease was unclear. The NGS approach applied permit not only the detection RNA viruses present in a sample, but also to determine their relative abundances. This approach can be used for a more comprehensive diagnosis of neonatal diarrhoea and is useful for the examination of the RNA virome in faeces.

## Methods

The study was conducted with samples selected from cases of neonatal diarrhoea (less than 7 days old) routinely submitted for diagnosis during 2017 to the Veterinary Diagnostic Laboratories of Infectious Diseases of the *Universitat Autònoma de Barcelona* (UAB, Barcelona, Spain) and *Universidad de León* (UL, León, Spain). Four samples from healthy non-diarrhoeic piglets were also included to be used as negative controls. In the cases studied, a bacteriological cause for the diarrhoea had been excluded after a microbiological culture for aerobes and anaerobes and molecular analyses (PCR for *E. coli* virulence factors and *C. perfringens* and *C. difficile* toxins). Only cases apparently unrelated (different farms with no evident connection) were included. Finally, 47 faecal samples, each one representing an outbreak of neonatal diarrhoea in 47 farms from *Catalonia*, *Castile and Leon*, *Aragon*, *Galicia* and *Valencia* were analysed (Additional file 1).

Faecal samples were kept frozen at − 80 °C until used. When needed, samples were thawed and diluted 1:5 in sterile distilled water. Then, to remove debris, diluted samples were sequentially clarified for 2 min at 2000 g, 5 min at 5000 g and 10 min at 10,000 g. Total RNA extraction was performed using the TRIzol reagent (ThermoFischer Scientific) following the manufacturer’s instructions. For each reaction, 250 μL of the diluted faeces was used.

The assessment of the diversity of viral RNA within each sample was directly characterised from the total RNA extracted, without any previous amplification step (no primers were required), applying a NGS approach previously applied to faeces [36]. Briefly, the procedures included: (i) the construction of a genomic library for Illumina NGS sequencing from the total RNA extracted, using a commercial protocol and reagents (Protocol for use with Purified mRNA or rRNA Depleted RNA and NEBNext® Ultra™ II RNA Library Prep Kit for Illumina®, New England Biolabs); ii) the NGS runs using an Illumina MiSeq platform available at the Genomics and Bioinformatics Service of the UAB and a read length of 250 bp; iii) the trimming of low quality reads (those showing a QC score < 20 as determined by FastQC©software, Babraham informatics), using Trimmomatic© [38]; iv) the taxonomic classification of the quality reads against a pre-built database containing the viral genomes available at GenBank with Kraken [39]; v) the initial filtering of quality reads against a reference file containing the concatenated sequences of a panel of RNA viruses described in faeces (Additional file 2); vi) the mapping of quality reads against the complete genomes of the RNA virus identified in the steps iv) and v), with the Burrows-Wheeler Aligner [40], applying the BWA-MEM algorithm for long reads [41]; and vii) the assembly of a consensus sequence in fasta format for every sample and RNA species identified, using the program QUASR [42]. For the software programs used in steps iii), iv), vi) and vii) - FastQC©software, Kraken, Burrows-Wheeler Aligner and QUASR, respectively -, the default parameters of every program were applied. Next, a database for every RNA species identified was constructed by downloading the available complete genome sequences from GenBank. Finally, the sequences obtained were aligned and phylogenetically compared with the datasets constructed for every complete genome, or segment in the segmented genomes identified. The phylogenetic relationships among sequences were analysed using the software MEGA7 [43], by means of a Neighbor-joining (NJ) algorithm, using the matrix of pairwise p-distances and 10,000 bootstrap replicates to estimate the confidence of the internal branches of the trees.

In the cases where a presumptive aetiological agent could not be clearly established, further analysis to detect unknown RNA viruses were undertaken with the program rnaSPAdes [44], an RNAseq de novo assembler included in the package SPADES [45]. Also, the RNAseq outputs were analysed with VirFind, a web-based bioinformatic pipeline specifically designed for virus detection and discovery [46]. In both cases, the default parameters provided by the programs were used.

## Supplementary information

**Additional file 1:** Summary of the 51 samples analyzed: name, origin, RNA viruses detected and number of reads indexed.

**Additional file 2:** Summary of the RNA viruses and Accession Numbers of the strains used to filter the quality reads in the step iv) of the NGS approach, used together with step iii), to identify the RNA virus species present in a given sample.

## Data Availability

The complete genome sequences obtained and analysed during the current study are available in the National Center for Biotechnology Information database repository, (https://www.ncbi.nlm.nih.gov/) with the Accession Numbers MH238075 to MH238338, MK936372 to MK936426, MK953017 to MK953236, and MK962320 to MK962342.

## Abbreviations

*AstV3*: *Porcine Astrovirus* type 3
*AstV4*: *Porcine Astrovirus* type 4
*AstV5*: *Porcine Astrovirus* type 5
BWA-MEM: Burrows-Wheeler Alignment tool with Maximal Exact Matches
*EntVG*: *Enterovirus* G
*KobuV*: *Kobuvirus*
NGS: Next Generation Sequencing
*PasiV*: *Pasivirus*
PCR: Polymerase Chain Reaction
*PDCoV*: *Porcine deltacoronavirus*
*PEDV*: *Porcine epidemic diarrhoea virus*
*PosaV*: *Posavirus*
QC: Quality Control score
RNA: Ribonucleic acid
*RVA*: *Rotavirus A*
*RVB*: *Rotavirus B*
*RVC*: *Rotavirus C*
*RVH*: *Rotavirus H*
*SAV*: *Sapovirus*
*TGEV*: *Transmissible gastroenteritis virus*
