# Supplementary material for: High levels of unreported intraspecific diversity among RNA viruses in faeces of neonatal piglets with diarrhoea
Source: BMC Vet Res. 2019 Dec 5;15:441. doi: 10.1186/s12917-019-2204-2 (PMC6896758; doi:10.1186/s12917-019-2204-2)
Supplement: Supplementary file 1 — Additional file 1: Summary of the 51 samples analyzed: name, origin, RNA viruses detected and number of reads indexed. [file 12917_2019_2204_MOESM1_ESM.docx]

**Additional file 1.** Summary of the 51 samples analyzed: name, origin, RNA viruses detected and number of reads indexed. Virus abbreviations: *Rotavirus A*, *RVA*; *Rotavirus B, RVB; Rotavirus C, RVC; Kobuvirus, KobuV; Sapovirus, SAV; Astrovirus 3, AstV3; Astrovirus 4, AstV4; Astrovirus 5, AstV5; Pasivirus, PasiV; Posavirus, PosaV; Porcine Epidemic Diarrhea Virus, PEDV; Enterovirus G, EntVG.* Result and viral load: Numbers in bold correspond to samples where whole genome was obtained), while numbers in italics correspond to samples where partial sequences were obtained. An asterisk indicates cases where *RVA* genomes were already published in [31]. The last four light grey lines correspond to the four negative (non-diarrheic) samples analysed.

| Sample | Origin | *RVA* | *RVB* | *RVC* | *KobuV* | *SAV* | *AstV3* | *AstV4* | *AstV5* | *PasiV* | *PosaV* | *PEDV* | *EntVG* | N |
| --- | --- | --- | --- | --- | --- | --- | --- | --- | --- | --- | --- | --- | --- | --- |
| VC20B | Castilla y León | **1.2·10e4** | **1.4·10e4** | **3.5·10e3** | **5.4·10e2** |  |  |  |  |  |  |  |  | 4 |
| VC7C | Castilla y León | **1.1·10e4** |  | **3.7·10e3** |  |  |  |  |  |  |  | *4.0·10e0* |  | 3 |
| P259C | Catalunya | **2.5·10e4** |  | **1.6·10e5** | **6.3·10e3** |  |  |  | *3.6·10e1* |  |  | *7.0·10e0* |  | 5 |
| P451* | Catalunya | **3.4·10e4** |  | *1.4·10e1* |  |  |  |  |  |  |  |  |  | 2 |
| P341* | Catalunya | **2.1·10e4** |  |  |  |  |  |  |  |  |  |  |  | 1 |
| P108* | Catalunya | **1.9·10e4** |  |  |  |  |  |  |  |  |  |  |  | 1 |
| P264* | Catalunya | **2.2·10e4** |  |  |  |  |  |  |  |  |  |  |  | 1 |
| P376* | Aragón | **2.4·10e4** |  |  |  |  |  |  |  |  |  |  |  | 1 |
| P348* | Catalunya | **2.8·10e4** |  |  |  |  |  |  |  |  |  |  |  | 1 |
| P447* | Catalunya | **3.1·10e4** |  |  |  |  | *8.2·10e1* |  |  |  |  |  |  | 2 |
| P37* | Catalunya | **5.6·10e4** |  |  | **6.6·10e2** |  |  |  |  |  |  |  |  | 2 |
| P222* | Catalunya | **1.7·10e4** |  |  | *1.1·10e2* |  |  |  |  |  |  |  |  | 2 |
| P471* | Catalunya | **2.7·10e4** |  |  | *1.2·10e1* |  |  |  |  |  |  |  |  | 2 |
| P452* | Catalunya | **6.3·10e4** |  |  | **2.1·10e4** | **6.6·10e2** |  |  |  |  |  |  |  | 3 |
| P284* | Catalunya | **5.1·10e4** |  |  | **1.6·10e4** | **3.5·10e4** |  |  |  |  |  |  |  | 3 |
| P255* | Catalunya | **3.3·10e4** |  |  | *9.4·10e2* | *6.0·10e0* |  |  |  |  |  |  |  | 3 |
| P456* | Castilla y León | **2.5·10e4** |  |  | *1.6·10e2* | *2.4·10e2* |  | *6.0·10e0* |  |  |  |  |  | 4 |
| P256C | Catalunya | **2.0·10e5** |  | **1.4·10e5** | *2.3·10e2* |  |  |  | *4.9·10e2* |  |  |  |  | 4 |
| VT29 | Castilla y León | **2.0·10e5** |  | *4.0·10e0* | *3.4·10e2* |  |  |  |  |  |  |  |  | 3 |
| P393ds* | Catalunya | **5.1·10e5** |  |  | **1.5·10e4** |  |  |  |  |  |  |  |  | 2 |
| P437ds* | Catalunya | **2.1·10e5** |  |  | **7.5·10e2** |  |  |  |  |  |  |  |  | 2 |
| P394ds* | Catalunya | **1.1·10e5** |  |  |  |  |  |  |  |  |  | *2.0·10e0* | *5.0·10e1* | 3 |
| P486* | Catalunya | **1.6·10e5** |  |  | *3.4·10e2* |  |  | *2.0·10e0* |  |  |  | *5.0·10e0* | *9.0·10e0* | 5 |
| VT25C | Castilla y León | *3.9·10e2* |  | **2.3·10e3** | **6.8·10e2** | *4.0·10e0* |  |  |  |  |  |  |  | 4 |
| VT11 | Castilla y León | *6.0·10e2* |  |  | *8.4·10e1* |  |  |  |  |  |  |  |  | 2 |

**Supplementary Material 1. Continued**

| Sample | Origin | *RVA* | *RVB* | *RVC* | *KobuV* | *SAV* | *AstV3* | *AstV4* | *AstV5* | *PasiV* | *PosaV* | *PEDV* | *EntVG* | N |
| --- | --- | --- | --- | --- | --- | --- | --- | --- | --- | --- | --- | --- | --- | --- |
| P1C | Castilla y León |  | **1.3·10e3** | **1.6·10e4** | *3.0·10e1* | T 10e0 | 2.0·10e0 | 1.0·10e1 |  |  |  |  |  | 6 |
| B378 | Aragón |  | **2.4·10e4** | *6.0·10e1* | *4.9·10e2* |  |  |  |  |  |  |  |  | 3 |
| B422 | Catalunya |  | **1.9·10e4** | *7.0·10e2* | *2.8·10e2* |  |  |  |  |  |  |  |  | 3 |
| B304 | Catalunya |  | **3.1·10e4** |  | **5.1·10e3** |  |  |  |  |  |  |  |  | 2 |
| B377 | Aragón |  | **2.5·10e4** |  | *1.1·10e2* |  | **5.6·10e2** |  |  |  |  |  |  | 3 |
| B333 | Catalunya |  | **1.7·10e4** |  | *5.4·10e2* |  | **2.5·10e3** |  |  |  |  |  |  | 3 |
| P2B | Catalunya |  | **3.0·10e4** |  | **1.2·10e3** |  | *6.0·10e0* |  |  |  |  |  |  | 3 |
| C257 | Catalunya |  |  | **1.1·10e3** | *1.3·10e1* |  |  |  |  |  |  |  |  | 2 |
| C247 | Catalunya |  |  | **4.7·10e5** | **6.3·10e2** |  |  |  |  |  |  |  |  | 2 |
| C429 | Catalunya |  |  | **1.3·10e5** | **4.5·10e2** |  |  |  |  |  |  |  |  | 2 |
| C375 | Catalunya |  |  | **1.0·10e5** | **1.3·10e4** | *1.2·10e2* |  |  |  |  |  |  |  | 3 |
| P461-C | València |  |  | **1.2·10e5** | **2.1·10e4** | *1.0·10e1* |  |  |  |  |  |  |  | 3 |
| 243A-2 | Catalunya |  |  | *2.7·10e3* | *2.5·10e2* |  |  |  |  |  |  |  |  | 2 |
| VT5 | Castilla y León |  |  | *5.0·10e0* | *2.3·10e2* |  |  |  |  |  |  |  |  | 2 |
| VC14 | Castilla y León |  |  | *7.0·10e0* | **9.3·10e3** |  |  |  |  |  |  | *6.0·10e0* |  | 3 |
| VC6 | Castilla y León |  |  | *6.0·10e0* | *2.9·10e2* | **2.0·10e2** | *2.0·10e0* | *8.0·10e0* | *1.1·10e2* |  |  | *7.0·10e0* |  | 7 |
| VC8 | Castilla y León |  |  |  | **8.9 10e3** |  |  |  |  |  |  |  |  | 1 |
| 378A-2 | Aragón |  |  |  | *3.4·10e1* |  |  |  |  |  |  |  |  | 1 |
| VC10 | Castilla y León |  |  |  | *2.8·10e2* |  |  |  |  |  |  |  |  | 1 |
| VC12 | Galicia |  |  |  | *2.0·10e0* |  |  |  |  |  |  |  |  | 1 |
| 361A-2 | Catalunya |  |  |  | *2.9·10e2* | **1.4·10e4** | *1.1·10e2* |  |  |  |  |  |  | 3 |
| VC11 | Castilla y León |  |  |  | **4.5·10e4** |  |  | *2.8·10e1* | *2.4·10e1* | *2.0·10e0* | *1.1·10e1* |  | *7.0·10e0* | 6 |
| 1DP | Catalunya |  |  |  | *7.0·10e0* |  |  |  |  |  |  |  |  | 1 |
| 2DP | Catalunya |  |  |  |  |  | *9.0·10e0* | *1.1·10e1* |  |  |  |  |  | 2 |
| 3DP | Catalunya | *2.0·10e0* | *1.0·10e0* |  |  |  | *1.6·10e1* |  | *5.6·10e1* |  |  |  | *2.0·10e0* | 5 |
| 4DP | Catalunya |  |  |  | *1.6·10e1* | *5.0·10e0* |  |  |  |  |  |  |  | 2 |
