# Supplementary material for: High levels of unreported intraspecific diversity among RNA viruses in faeces of neonatal piglets with diarrhoea
Source: BMC Vet Res. 2019 Dec 5;15:441. doi: 10.1186/s12917-019-2204-2 (PMC6896758; doi:10.1186/s12917-019-2204-2)
Supplement: Supplementary file 2 — Additional file 2: Summary of the RNA viruses and Accession Numbers of the strains used to filter the quality reads in the step iv) of the NGS approach, used together with step iii), to identify the RNA virus species present in a given sample. [file 12917_2019_2204_MOESM2_ESM.docx]

**Additional file 2.** Summary of the RNA viruses and Accession Numbers of the strains used to filter the quality reads in the step iv) of the NGS approach, used together with step iii), to identify the RNA virus species present in a given sample.

| RNA virus | Accession Number |
| --- | --- |
| *Porcine Rotavirus A* | KU363134 |
| *Porcine Rotavirus B* | KR052718 |
| *Porcine Rotavirus C* | KP982883 |
| *Porcine Rotavirus H* | KU254587 |
| *Porcine Astrovirus type 2* | MG930777 |
| *Porcine Astrovirus type 3* | JX556691 |
| *Porcine Astrovirus type 4* | LC201602 |
| *Porcine Astrovirus type 5* | JX556693 |
| *Kobuvirus* | MH184664 |
| *Swine Pasivirus* | JQ316470 |
| *Posavirus* | JF713721 |
| *Sapelovirus* | LT900497 |
| *Porcine Sapovirus* | KT922087 |
| *Teschovirus* | AB049536 |
| *Transmissible gastroenteritis virus* | DQ811788 |
| *Porcine Torovirus* | LT900503 |
| *Porcine enterovirus 1* | AJ011380 |
| *Porcine enterovirus 8* | AF406813 |
| *Porcine enterovirus 9* | AF363453 |
| *Porcine enterovirus 10* | AF363455 |
| *Porcine enterovirus 15* | JN807387 |
| *Porcine enterovirus G* | KF985175 |
| *Porcine Alphacoronavirus* | MF370205 |
| *Porcine Deltacoronavirus* | MF431743 |
| *Hepatitis E* | KT581448 |
| *Porcine Epidemic Diarrhea Virus* | KM189367 |
